# Antioxidant Activities in the Hemolymph and Fat Body of Physiologically and Prematurely Aging Bees (*Apis mellifera*)

**DOI:** 10.3390/antiox14040373

**Published:** 2025-03-21

**Authors:** Magdalena Kunat-Budzyńska, Patrycja Staniszewska, Krzysztof Olszewski, Aneta Strachecka

**Affiliations:** 1 Department of Immunobiology, Institute of Biological Sciences, Faculty of Biology and Biotechnology, Maria Curie-Skłodowska University, Akademicka 19, 20-033 Lublin, Poland; 2 Department of Invertebrate Ecophysiology and Experimental Biology, University of Life Sciences in Lublin, 20-950 Lublin, Poland; patrycja.staniszewska@up.lublin.pl; 3 Subdepartment of Apidology, Institute of Biological Basis of Animal Production, Faculty of Animal Sciences and Bioeconomy, University of Life Sciences in Lublin, 20-950 Lublin, Poland; krzysztof.olszewski@up.lublin.pl

**Keywords:** antioxidant, fat body, hemolymph, honeybee aging, workers, *Varroa destructor*

## Abstract

Aging is a multifactorial process that occurs in all living organisms, including bees. One of the factors accelerating this process is stress caused in bees by *Varroa destructor*. The research aim was to compare antioxidant system activities in different tissues and in different fat body segments (sternite, tergite 3 and 5) in workers aging naturally (physiologically) and prematurely (affected by *V. destructor*). The CAT, GPx, GST, and SOD activities in naturally aging workers were higher in all the tissues/fat body segments and age groups compared to prematurely aging workers. These antioxidant activities increased with age, reaching a maximum at 21 (in tergite 3 and sternite) or 28 days of age (in the hemolymph and tergite 5) in naturally aging workers, and then decreased in the oldest ones (at 35 days of age). In the prematurely aging workers, the antioxidant activities in the fat body decreased along with age. The highest activities were identified in the fat body of tergite 5, which may suggest its role in detoxification processes. Our results are a starting point for a better understanding of the mechanisms related to oxidative stress, aging, and their correlation with the health and lifespan of bees.

## 1. Introduction

Aging is a natural biological process that is characterized by a decrease in the efficiency of processes occurring in cells, tissues, and organs, which consequently leads to many diseases and death. We can distinguish two models of aging: the first is adaptive aging, which assumes that the aging process is genetically controlled, and the second is physiological aging, also described as uncontrolled (non-programmed) aging. The latter has become one of the key areas of research in recent years due to the growing number of older people in society and the need to improve the quality of life in old age [1].

Current knowledge of the aging process is based on experiments using model organisms characterized by short life cycles, such as *Drosophila melanogaster*, *Caenorhabditis elegans*, *Saccharomyces cerevisiae*, or mice. When the bee genome was sequenced in 2006, revealing that it shares many genes with humans, including genes encoding enzymes related to metabolism and components of the innate immune system, it started to be used in studies to better understand the mechanisms of aging [2,3]. Firstly, research on bees, as invertebrates, is not subject to ethical restrictions as with vertebrates, which in turn reduces the costs associated with conducting basic research [3,4]. Secondly, honeybees exhibit double polymorphism, which on the one hand is characterized by the presence of a long-lived queen and short-lived workers, while on the other hand, it classifies workers depending on the roles they perform [1,5,6]. A very important phenomenon occurring in bees is the reversion of worker life cycles, which is manifested by the ability to change the roles performed and reverse the physiological aging processes under certain conditions. For example, foragers can become nurses when the number of larvae in the hive exceeds the number of nurses. In turn, the absence of foragers due to starvation or pathogen infections accelerates the maturation of young bees, which take over their tasks [6,7,8,9,10,11,12]. During the transition between the roles of nurses and foragers, workers undergo significant physiological changes. These include changes in hormone levels, metabolic rate, gene expression, signaling mechanisms, nutrient storage, e.g., of the vitellogenin protein, and stress resistance [7,12]. For example, during flight, foragers consume more energy, as a result of which they show an almost 100-fold increase in metabolic rate compared to hive-dwelling bees. As a consequence, foragers may produce reactive oxygen species due to their enormous energetic and metabolic effort, which leads to oxidative stress, which in turn accelerates the aging process [1,13,14]. Thirdly, honeybees are similar to vertebrates, among other things, in terms of organ function, i.e., the fat body plays a role analogous to the liver, pancreas, or adipose tissue in mammals [3,15].

The fat body in bees is located inside the body, filling the spaces in the organism. The fat body can take two forms: the first is the perivisceral layer, which surrounds the internal organs, e.g., the intestines or reproductive system, and the second is the subcuticular layer, which is located close to the inner side of the cuticle [16,17,18]. It consists of several types of cells, which include chromatocytes, mycetocytes, oenocytes, trophocytes, and urocytes. The fat body of winter bees (with a lifespan up to 250 days) compared to summer workers (with a shorter lifespan of 25 to 40-days) is characterized by increased volume, trophocyte, and oenocyte sizes, and metabolism due to endothermic heat production (thanks to which, they can survive at low temperatures), as well as large nutrient reserves, consisting mainly of proteins and lipids [19,20]. The fat body performs many functions in bees, being primarily responsible for key metabolic processes, including the synthesis of proteins, lipids and glycogen, and energy storage [15,16,20,21,22]. It is also an endocrine organ, as it produces many immune peptides, e.g., the antimicrobial peptides (AMPs) [16,23]. In addition, various compounds, including those with antioxidant properties, are produced in different fat body locations/segments and then released into the hemolymph [22].

Ramsey et al. [24] showed that *Varroa destructor*, which is the main cause of the shortening of the expected lifespan of bees and consequently the acceleration of their aging processes and the decline in their population, feeds directly on the apian fat body, not on the hemolymph. However, since that study, it has not been shown what effect infestation with mites will have on the functioning of the subcuticular fat body, which is segmental. Therefore, our research fits into this scientific discourse. These authors have shown that mites on the metasoma are found with the greatest frequency (88.5%) underneath the sternite or tergite of the third metasomal segment. Strachecka et al. [18] revealed that the fat body from these locations has the highest metabolic activities. Therefore, the material for our study was from the fat body of the sternite, tergite 3, and tergite 5. In addition, it is known that the parasite causes a reduction in the number of hemocytes and an increase in the level of reactive oxygen species in the hemolymph, reduces the volume of the fat body, and thus weakens the immune system of adult bees [14,24,25,26,27,28]. Moreover, *V. destructor* infection leads to significant reductions in protein and antioxidant enzyme levels in developing bee larvae [29]. Antioxidant enzymes provide protection against oxidative stress, for example, by neutralizing harmful radicals and oxidants generated during parasitic infections.

Hence, we hypothesized that workers whose aging processes are accelerated and their lifespan shortened as a result of parasitization by *V. destructor* are characterized by reduced activities of the antioxidant enzymes catalase (CAT), glutathione S-transferase (GST), glutathione peroxidase (GPx), and superoxide dismutase (SOD), and reduced levels of total antioxidant capacity (TAC) in various tissues (hemolymph vs. fat body) and in different fat body locations (sternite, tergite 3, and tergite 5). The aim of our study was to compare antioxidant levels in various tissues and different fat body locations in naturally (physiologically) and prematurely aging (*V. destructor*-affected) workers.

## 2. Materials and Methods

### 2.1. Obtaining 1-Day-Old Bees

Eight colonies kept (in Dadant beehives) in an apiary that belongs to the University of Life Sciences in Lublin (51°22′ N, 22°63′ E), Poland provided one-day-old bees for the experiments. The colonies were made up of healthy bees. A queen-excluder comb-cage was used to house the queens for 12 hours in confinement with one void comb for laying eggs in it. In twenty days’ time following the queens having laid their eggs, the combs were moved to an incubator, in which the 1-day-old workers emerged. Thirty freshly emerged workers were collected for laboratory analyses. The remaining workers were marked with different colors (POSCA PC-3M marker, Uni Mitsubishi Pencil, Shinagawa, Tokyo, Japan; 6000 workers) separately and randomly placed in six colonies (in mini beehives) with small frames (210 mm × 170 mm). The colonies were previously prepared in such a way that the workers and brood were healthy and free from *V. destructor* in three of them, and this group was treated as the control group in which the workers aged normally/physiologically. Bees not infected by *Varroa* came from colonies successfully treated against varroosis. The same treatment was applied to colonies not infected to which bees were introduced. Therapeutic treatments were carried out in autumn in October and in spring in March after bringing the colony to a brood-free state through prior isolation of the queen. In such a short time, mites would not have been able to multiply to a point where they could strongly infest the colony. In addition, during the collection of bees for testing, each individual was observed to see if there were any parasites on the body and between the segments of the abdomen. In contrast, in the other three colonies, the workers and brood had mites, and this group was treated as infested/diseased, with the bees aging prematurely. The marked bees from the colonies free of *V. destructor* were collected on the 14th, 21st, 28th and 35th day of age (3 colonies × 4 samplings × 10 bees); while in the mite-infested colonies, only the 14- and 21-day-old workers that had *V. destructor* mites on them were collected, as the bees did not survive until the next samplings (3 colonies × 2 samplings × 10 bees). In each sampling, 10 workers were collected from each colony of each of the two experimental groups. This allowed for obtaining a representative number of bee samples. A total of 210 workers were collected for the study.

### 2.2. Laboratory Analyses

#### 2.2.1. Hemolymph and Fat Body Collection

**Figure 1:**
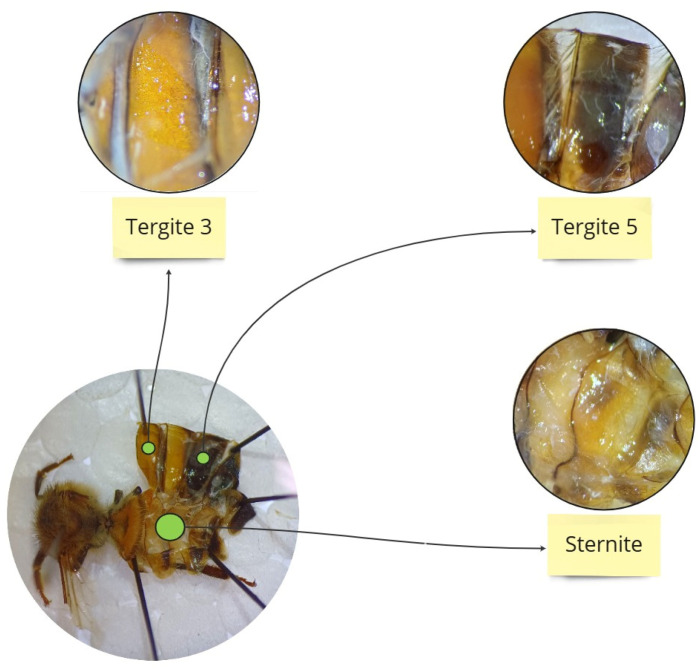
Location of the individual segments from which the fat body was collected.

To obtain fresh hemolymph, a glass capillary (20 µL, “end to end” type, without anticoagulant; Medlab Products, Raszyn, Poland) was individually inserted between the third and fourth tergites of a living worker as per Łoś and Strachecka [30]. The hemolymph volumes were separately measured in each capillary. Hemolymph from an individual bee was collected in one sterile Eppendorf tube containing 25 µL of ice-cooled 0.6% NaCl. The hemolymph solutions were immediately refrigerated at −40 °C for further biochemical analyses. Subsequently, the individual insects were thawed gradually and the fat body from the third and fifth tergites and the sternite was prepared according to the methodology described by Bryś et al. [31]. The choice of these three locations for biochemical analyses was based on previous studies by Strachecka et al. [18], which demonstrated that the fat body from these locations is metabolically the most active (Figure 1). Next, the tissues were manually homogenized and centrifuged at 4 °C for 1 min at 3000× *g*. The supernatants were frozen at −25 °C for further biochemical analyses.

#### 2.2.2. Biochemical Analyses

The following antioxidants were assayed in the hemolymph solutions and fat body supernatants.

Catalase (CAT) activities using the method specified in a commercial catalase assay kit from Cayman Chemical Company, East Ellsworth Road, Ann Arbor, MI, USA; 707002.

Glutathione peroxidase (GPx) activities using the method specified in a commercial glutathione peroxidase assay kit from Sigma Aldrich, Schnelldorf, Germany; MAK437-1KT.

Superoxide dismutase (SOD) activities based on the method specified in a commercial SOD assay kit from Sigma Aldrich, Schnelldorf, Germany; 19160-1KT-F.

Glutathione S-transferase (GST) activities following the method explained in the commercial glutathione S-transferase assay kit from Sigma Aldrich, Schnelldorf, Germany; MAK 435-1KT.

Total antioxidant capacity (TAC) employing the method described in a commercial antioxidant assay kit from Cayman Chemical Company, East Ellsworth Road Ann Arbor, MI, USA; 709001.

The antioxidant enzyme activities were calculated per 1 mg of protein. All the tested parameters were measured using a spectrophotometer [22].

### 2.3. Statistical Analyses

The results were analyzed statistically using Statistica software, version 13.3 (2017) for Windows, StatSoft Inc., Tulsa, OK, USA. Data distribution was checked using the Shapiro–Wilk test. The effects of the tissue/fat body location (hemolymph and the fat body from tergite 3, tergite 5, and sternite) in each age group (n  =  30 bees) on CAT, GPx, GST and SOD activities and TAC levels were measured with the Kruskal–Wallis test. The effects of age (1, 14, 21, 28 and 35-day) on CAT, GPx, GST, and SOD activities and TAC levels for the particular tissues/fat body locations (hemolymph and the fat body from tergite 3, tergite 5, and sternite) were assessed in a similar fashion. For each tissue/fat body location, CAT, GPx, GST, and SOD activities and TAC levels were compared between the age groups with the Mann–Whitney U test.

## 3. Results

With the exception of GPx activities in the control group, the effects of tissue/fat body location (hemolymph or the fat body from tergite 3, tergite 5, and sternite) were statistically significant (Table 1).

The age of the workers (1, 14, 21, 28, and 35 days) had a statistically significant effect on the activities of antioxidant enzymes and TAC levels (Table 2).

### 3.1. Activities of Antioxidant Enzymes: CAT, GPx, GST, and SOD

**Figure 2:**
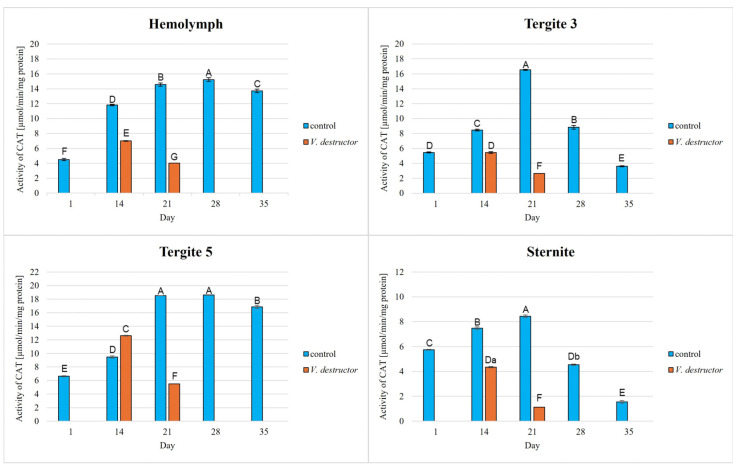
Catalase (CAT) activities in the hemolymph and the fat body from tergite 3, tergite 5, and sternite of the naturally (physiologically) aging 1-, 14-, 21-, and 35-day-old workers and in the prematurely aging 14- and 21-day-old workers (affected by *V. destructor*). A, B, C, D, E, F, G—capital letters indicate statistically significant differences between the groups at *p* ≤ 0.01. a, b—lowercase letters indicate statistically significant differences between the groups at *p* ≤ 0.05.

**Figure 3:**
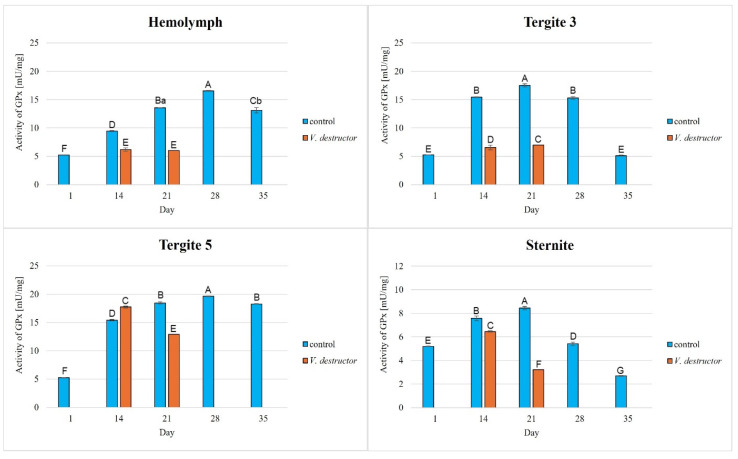
Glutathione peroxidase (GPx) activities in the hemolymph and the fat body from tergite 3, tergite 5, and the sternite of the naturally (physiologically) aging 1-, 14-, 21-, and 35-day-old workers and in the prematurely aging 14- and 21-day-old workers (affected by *V. destructor*). A, B, C, D, E, F, G—capital letters indicate statistically significant differences between the groups at *p* ≤ 0.01. a, b—lowercase letters indicate statistically significant differences between the groups at *p* ≤ 0.05.

**Figure 4:**
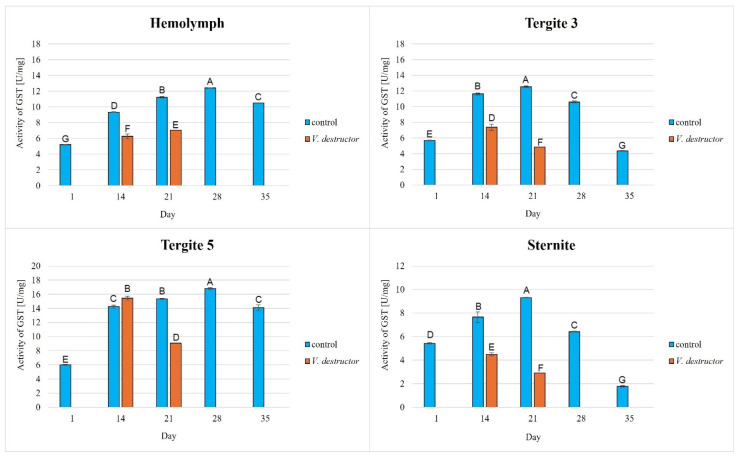
Glutathione S-transferase (GST) activities in the hemolymph and the fat body from tergite 3, tergite 5, and the sternite of the naturally (physiologically) aging 1-, 14-, 21-, and 35-day-old workers and in the prematurely aging 14- and 21-day-old workers (affected by *V. destructor*). A, B, C, D, E, F, G—capital letters indicate statistically significant differences between the groups at *p* ≤ 0.01.

**Figure 5:**
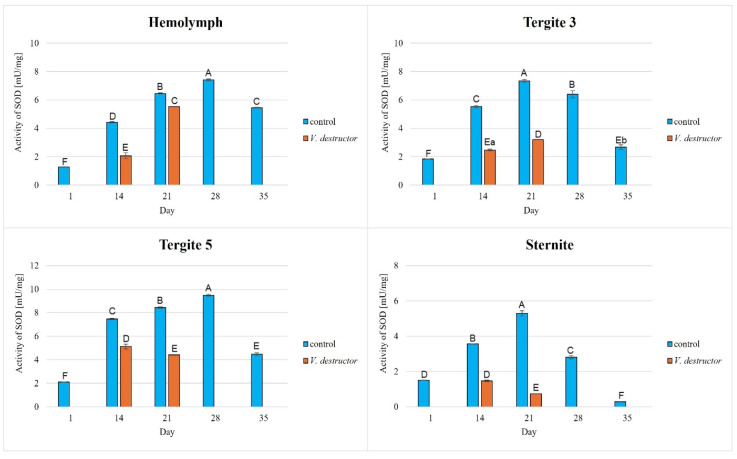
Superoxide dismutase (SOD) activities in the hemolymph and the fat body from tergite 3, tergite 5, and the sternite of the naturally (physiologically) aging 1-, 14-, 21-, and 35-day-old workers and in the prematurely aging 14- and 21-day-old workers (affected by *V. destructor*). A, B, C, D, E, F—capital letters indicate statistically significant differences between the groups at *p* ≤ 0.01. a, b—lowercase letters indicate statistically significant differences between the groups at *p* ≤ 0.05.

The activities of CAT, GPx, GST, and SOD increased with age, reaching a maximum in the 21-day-old (the fat body from tergite 3 and the sternite) and 28-day-old (the hemolymph and the fat body from tergite 5) naturally (physiologically) aging workers, and then decreased in the oldest (35-day-old) workers (Figure 2, Figure 3, Figure 4 and Figure 5).

The CAT, GPx, GST, and SOD activities in naturally (physiologically) aging workers were statistically significantly higher in all tissues/fat body locations and age groups compared to the (affected by *V. destructor*) prematurely aging workers (Figure 2, Figure 3, Figure 4 and Figure 5). The exceptions were the activities of CAT, GPx, and GST in the fat body of tergite 5 in the 14-day-old bees, where higher values were observed in those infected with *V. destructor* than in those from the control group (Figure 2, Figure 3 and Figure 4).

As regards the prematurely aging workers (affected by *V. destructor*), CAT, GPx, GST, and SOD activities were significantly higher in all the tissues/locations in the 14-day-old workers in comparison with the 21-day-old workers (Figure 2, Figure 3, Figure 4 and Figure 5). Hemolymph was an exception, where the opposite trend was noted for the GST and SOD activities, and the GPx activity remained at a similar level both in the 14- and 21-day-old workers (Figure 3, Figure 4 and Figure 5).

### 3.2. Levels of Total Antioxidant Capacity (TAC)

**Figure 6:**
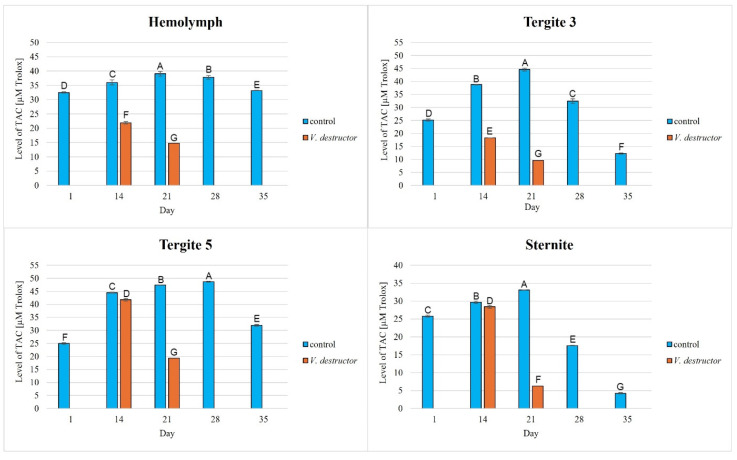
Total antioxidant capacity (TAC) levels in the hemolymph and the fat body from tergite 3, tergite 5, and the sternite in the naturally (physiologically) aging 1-, 14-, 21-, and 35-day-old workers and in the prematurely aging 14- and 21-day-old workers (affected by *V. destructor*). A, B, C, D, E, F, G—capital letters indicate statistically significant differences between the groups at *p* ≤ 0.01.

The TAC levels in the naturally (physiologically) aging workers were statistically significantly higher in all the tissues/fat body locations and age groups compared to the prematurely aging workers (affected by *V. destructor*). The TAC levels increased along with the age of the workers in the control group until the 21st (in the hemolymph and the fat body from tergite 3 and the sternite) or the 28th day of age (in the fat body from tergite 5), and then these values decreased. The highest values of TAC in this group were identified in the fat body from tergite 5 of the workers from day 14 to day 28 of their life. The TAC levels in the oldest workers were the highest in the hemolymph and the lowest in the fat body from the sternite (Figure 6). As far as the prematurely aging workers (affected by *V. destructor*) are concerned, the highest TAC levels were observed in the fat body from tergite 5 in the 14-day-old workers. In a similar way to the CAT activities in the 14-day-old prematurely aging workers, the TAC levels in each tissue/location were higher than in the 21-day-old workers. The age-related trend in the TAC levels was similar to that of the antioxidant enzymes, the exception being the hemolymph of the uninfected bees, where the TAC levels fluctuated within a small range.

## 4. Discussion

Antioxidant enzymes, such as catalase (CAT), superoxide dismutase (SOD), glutathione S-transferase (GST), and glutathione peroxidase (GPx), as well as the total antioxidant capacity (TAC) in bees are commonly used to monitor their oxidative stress and vitality and immunity levels. In our study, we focused on the analysis of the antioxidant system of bees in the context of aging processes. Antioxidant activities have been primarily described in apian hemolymph [32], entire bees [33] or in thoraces or/and abdomina or/and heads [14,34,35,36] in the context of the influence of anthropogenic factors, e.g., electromagnetic waves [37], monodiets [38], pesticides [39,40], or stimulators [41], etc. Only a few publications present information on the antioxidant system in the fat body of bees. Santos et al. [42] presented the expression of antioxidant genes (CuZnSOD, MnSOD, Gst1, catalase and GSH/GSSG) in the fat body of worker and queen larvae. Brejcha et al. [20] reported the expression of antioxidant genes in fat body cells of short-lived summer and long-lived winter workers. Hsu and Hsieh [43] characterized CAT, GPx and SOD activities in trophocytes and other fat body cells of 1- and 50-day-old workers. Strachecka et al. [22] compared the activities of antioxidants in different locations of the subcuticular fat body in different castes of bees—workers, queens and rebels—just after their emergence. Bryś et al. [38] compared the activities of antioxidant enzymes in 1-, 7-, and 14-day-old worker bees fed monodiets in cage conditions. This publication supplements the knowledge of the physiology of the fat body with the following information: (1) profiles of the antioxidant activities in different segments/locations of the fat body not only in 1- to 14-day-old workers, but also in older ones, even those 35 days of age; (2) comparisons of the activities of antioxidants in naturally/physiologically aging bees and those aging prematurely due to *V. destructor* infestation; (3) presentation of the effects of *V. destructor* on the activities of the above enzymes in the fat body from tergite 3, tergite 5, and the sternite and in the hemolymph of workers; (4) description of the antioxidant activities in the hemolymph and fat body of bees kept in hives, not in cage conditions, like in most publications.

### 4.1. Antioxidant Activities in Naturally/Physiologically Aging Bees

Physiological aging of bees begins when they transition to foraging, i.e., around day 18–21 of worker life [44,45]. At this age, workers fly out of the hive in search of nectar and pollen, and are therefore more exposed to external factors, including pathogens and pesticides that cause oxidative stress in them. In addition to these factors, workers use a lot of energy during flight, which causes an acceleration of their metabolism and in consequence leads to the production of ROS [14,46]. In the later stages of foraging, such features of worker aging can be observed as mechanical damage, weakened immunity, and damage due to oxidative stress in the optic lobes [47]. In order to limit the negative effects of oxidative stress, workers activate their antioxidant defenses, which include antioxidant enzymes such as CAT, GST, GPx, and SOD.

Our studies revealed that the activities of antioxidant enzymes increase with age, reaching a maximum on the 21st or 28th day (i.e., at an age when they are outside the hive foraging) depending on the tissue/fat body location and the enzyme type. It is worth noting here that this is currently the only publication that characterizes the activity of the antioxidant system not only in the hemolymph but also in various locations/segments of the fat body of old workers. Both Strachecka et al. [22] and Bryś et al. [38] presented the activities of these enzymes only in young bees at ages between 1 and 14 days that were kept in cages, i.e., in artificial conditions created for the purposes of the experiment. Therefore, our results are all the more valuable, as they illustrate the activity of the antioxidant system in natural hive conditions. Strachecka et al. [48] showed that the activities of CAT and GPx in the hemolymph are the highest in healthy 28-day-old workers, while the activities of SOD and GST are the highest at age 21 days. In turn, Skowronek et al. [49] showed that the SOD and GST activities in the hemolymph were the highest in the 28-day-old workers and CAT and GPx in the 21-day-old insects. The antioxidant activities in the bees in our experiment reached their maximum values in the 28-day-old naturally (physiologically) aging workers. The one-week difference in the highest-activity values of these enzymes between our workers and those in the study by Strachecka et al. [48] and Skowronek et al. [49] may be due, among other things, to environmental conditions, bee genetics, or other factors influencing the antioxidant system. The bees leaving the hive environment as well as the cage conditions [48,49] were associated with a decrease in the activity of the antioxidant system in the hemolymph of the workers (which was observed at age 35 days). Moreover, similarly to the experiment of Bryś et al. [38], we showed that the values of all the antioxidants in the bees up to 14 days of age systematically increased not only in the hemolymph but also in the individual segments/locations of the fat body, with the highest activities of GST and SOD identified in tergite 5. It is worth noting that this tendency was observed both in the bees kept in cage conditions [38] and in hives (Figure 2, Figure 3, Figure 4 and Figure 5). The activities of the enzymes closely relate to the function/metabolism of particular organelles in the fat body cells as well as ROS neutralization reactions. As reported by Scofield and Amdam [50], nurse bees retain elevated levels of lipids and other substances in the abdomen, also in the fat body, while forager bees contain very low levels of these compounds. This is a pattern that is likely to contribute to the efficacious performance of their social roles, which upholds the colony in an appropriate condition. This not only stems from the evolution of eusocial organisms [50] but also from the adaptation to volatile environmental conditions [51] and disease influence (as in the case of *Varroa*).

The TAC levels, as shown in publications by Słowińska et al. [40], Strachecka et al. [52,53], and Skowronek et al. [49], increased in the hemolymph of workers along with their aging processes until the 21st–30th day of age and then decreased. Hence, the antioxidant system works the most effectively in bees that have just turned foragers and are more exposed than nesting bees to harmful environmental factors causing oxidative stress. This reduction in TAC in flying/old bees, as shown by Margotta et al. [14], is the result of a decreased efficiency of mitochondrial respiration and electron transport chain activity, which results in early metabolic and behavioral senility [54]. As foragers age, the synthesis of many compounds (e.g., glycogen) slows and even eventually ceases, suggesting that multiple metabolic pathways (e.g., carbohydrates, lipids, etc.) are damaged. As a result, ROS accumulate to be then neutralized by the antioxidant system. Antioxidant consumption is conducive to low TAC levels.

The lowest antioxidant enzyme activities and TAC levels were noted in the oldest workers (35 days old), which were at an advanced stage of life and showed signs of aging (Figure 2, Figure 3, Figure 4, Figure 5 and Figure 6). They are characterized by greater sensitivity to food deficiency, temperature changes, and oxidative stress [55]. The aging rate of bees may to a large extent be influenced by access to the food base and environmental pollution, including the presence of pesticides and heavy metals, which accelerate the aging process. Precisely determining the influence of these factors on the aging processes in bees will allow for a thorough understanding of the biochemical, physiological, genetic, and epigenetic mechanisms occurring in bees as they age.

### 4.2. Antioxidant Activities in Prematurely Aging Bees (Affected by V. destructor)

There is little information on the effect of *V. destructor* infestation on the activities of the antioxidant enzymes as one of the factors in the aging processes in bees. It is known that this ectoparasitic mite causes a shortening of the lifespan of bees by feeding on the fat body and hemolymph [24,56], among other factors, its salivary proteins damage bee hemocytes and alter the profile of apian proteins and other metabolically important compounds, and the mite is a vector of viruses [56]. In our study, the workers with a shortened lifespan due to *V. destructor* survived only 21 days compared to those aging naturally (physiologically), which survived 35 days. This age was also reported by Morfin et al. [56] for workers that had one mite on them. However, in the case of workers that emerged with two or three mites, their life expectancy was only 8.5 days. Additionally, we observed that the activities of the antioxidant enzymes were lower in the prematurely aging workers (affected by *V. destructor*) than in the normally aging (healthy) bees and tended to decrease along with their age (Figure 2, Figure 3, Figure 4 and Figure 5). Gülmez et al. [26] showed that homogenates from entire workers infested with *V. destructor* are characterized by higher SOD activities and lower CAT and GST activities compared to healthy workers. In turn, Łopieńska-Biernat et al. [57] showed that *V. destructor*-infested feral bees from tree holes had lower SOD activities and higher CAT and GST activities compared to uninfested workers. The above results of these two publications are burdened with error and present the activity of the antioxidant system in whole insects (bee homogenates) taking into account the entire microflora that is on them (on the cuticle) and in them, and perhaps also the mites that were present on the bees. Farjan et al. [58], Badotra et al. [29], and Lipiński and Żółtowska [59] also showed increased antioxidant activity and signs of oxidative stress in homogenates of various preimaginal stages of bees infested with mites, indicating this as the main cause of developmental defects and increased brood mortality. By characterizing the antioxidant system in terms of tissue specificity (hemolymph vs. fat body) and taking into account the segmentation of the fat body, we avoided errors related to the species affiliation (bee vs. microorganisms vs. mite) of the enzymes. In this respect, our publication is innovative and fits into the contemporary scientific discourse on premature aging under the influence of unfavorable factors. Reduced antioxidant system activities in all tissues/fat body locations of bees infested with *V. destructor* is most likely a consequence of mites feeding on the fat body of bees, as a result of which this tissue loses its structure and consequently its functions [24]. Our studies confirm that infested bees lose their antioxidant and detoxification capacities in the fat body and hemolymph. As a result, they most likely accumulate ROS, which damage proteins, lipids, sugars, and other structural and metabolic compounds of bee cells. The lack or loss of the fat body in a bee leads to disorders in the synthesis, activation, and functioning of many compounds, including the antioxidant system, which leads to immunological disorders and accelerated aging processes and ultimately the death of the insect. The effects of oxidative stress, as reported by Farjan et al. [58,60], can be delayed, even in bees infested with *V. destructor*, by adding antioxidants to the diet, e.g., vitamin C or other stimulants (e.g., CBD, CoQ-10, caffeine, curcumin) [48,49,53,61].

### 4.3. Antioxidant Activities in Different Tissues/Fat Body Locations

Various authors, including Gülmez et al. [26] and Orcic et al. [62], have determined antioxidant activities, usually in homogenates of whole bees. Hsu et al. [43] have found fat body CAT activities to increase with age, while SOD activities were observed to decrease. Unfortunately, these authors did not specify the part of the fat body sampled (visceral or subcuticular) or its location (segment), hence the difference between our and their results. In our study, we focused on determining the activity of the antioxidant system in the hemolymph and in individual segments of the subcuticular fat body, i.e., tergite 3, tergite 5, and the sternite, while taking into account the natural (physiological) and accelerated aging of workers. We corroborated the findings of Strachecka et al. [22] showing that antioxidant activities vary depending on the type of tissue (hemolymph vs. fat body) and fat body segments/locations (tergite 3, tergite 5, and sternite). These authors showed that the SOD and CAT activities were always the highest in the sternite, while the TAC levels were the highest in tergite 3 in different castes/subcastes of the 1-day-old insects. In our experiment, we showed that the antioxidant levels were the lowest in the fat body from the sternite or tergite 3, while being the highest in tergite 5. This is consistent with the results obtained by Bryś et al. [38], who suggested that the fat body assumes different physiological functions in individual segments/locations. The fat body from tergite 3 and the sternite is responsible for the accumulation of energy compounds, and that from tergite 5 for antioxidant and detoxification mechanisms [18,22,38]. The sternite and tergite 3 fat bodies are composed primarily of many large trophocytes, whose main function is to store glucose, glycogen, triglycerides, and other nutrients [18,22,63,64,65]. Furthermore, lower values of the antioxidant system in bees with a shortened lifespan (Figure 2, Figure 3, Figure 4, Figure 5 and Figure 6) are most likely the result of a decrease in the volume of the fat body (from the sternite and tergite 3) after being fed on by *V. destructor* [24]. What distinguishes the tergite 5 fat body is the large number of oenocytes [18], which, as reported by Huang et al. [65], are responsible for detoxification and are an analogue of the mammalian liver. Hence, it can be concluded that the fat body from tergite 5 shows the best predispositions to neutralize ROS, which in turn can prevent the effects of oxidative stress and premature aging of the organisms.

## 5. Conclusions

This study is the first report comparing antioxidant activities in different tissues/fat body locations in naturally (physiologically) aging workers and prematurely aging ones (affected by *V. destructor*) at different ages. *V. destructor* infestation leads to decreased activities of enzymes that are crucial for the functioning of the antioxidant system. This results in the accumulation of reactive oxygen species that cause damage at the cellular level, which in turn accelerates aging and shortens lifespan. The antioxidant system is the most active in the fat body from tergite 5. This location shows the best predisposition to neutralize ROS, which in turn can prevent the effects of oxidative stress and premature aging of the organisms. Mites sucking out the fat bodies, mainly from the sternite and tergite 3, contribute to their dysfunction and further reduce the antioxidant activities. Understanding the functions of individual fat body segments in bees will allow for a better understanding of their role in mechanisms related to oxidative stress and their correlation with bee health and longevity. Thus, this will help protect bees from environmental stress and support their health in the difficult conditions of the modern world. Gerontology is a field of science that allows us to better understand what factors and mechanisms underlie the complex processes of aging and to learn methods of delaying it, as in the context of bees as model organisms. Such studies have a practical aspect, because they will enable the creation of effective strategies for protecting these valuable pollinators, e.g., by enriching their diet with antioxidants or using preparations that strengthen their immunity, we can provide them with increased protection against pathogens, improve their subsistence conditions, and minimize/delay the effects of aging.

## Figures and Tables

**Table 1:** Effects of tissue/fat body location (hemolymph or the fat body from tergite 3, tergite 5, and sternite) in physiologically and prematurely (*V. destructor*-infested) aging workers on the activities of CAT, GPx, GST, SOD and TAC levels.

Group	Age (Days)	CAT	GPx	GST	SOD	TAC
control	1	H = 106.88 *p* = 0.000	H = 6.10 *p* = 0.106	H = 100.31 *p* = 0.000	H = 111.57 *p* = 0.000	H = 73.56 *p* = 0.000
control	14	H = 109.64 *p* = 0.000	H = 100.37 *p* = 0.000	H = 111.58 *p* = 0.000	H = 111.57 *p* = 0.000	H = 108.24 *p* = 0.000
*V. destructor*	14	H = 111.58 *p* = 0.000	H = 73.31 *p* = 0.000	H = 111.15 *p* = 0.000	H = 105.36 *p* = 0.000	H = 111.57 *p* = 0.000
control	21	H = 112.44 *p* = 0.000	H = 112.07 *p* = 0.000	H = 112.22 *p* = 0.000	H = 111.07 *p* = 0.000	H = 112.02 *p* = 0.000
*V. destructor*	21	H = 110.33 *p* = 0.000	H = 111.57 *p* = 0.000	H = 97.80 *p* = 0.000	H = 109.06 *p* = 0.000	H = 104.98 *p* = 0.000
control	28	H = 110.64 *p* = 0.000	H = 110.63 *p* = 0.000	H = 110.63 *p* = 0.000	H = 110.63 *p* = 0.000	H = 110.63 *p* = 0.000
control	35	H = 111.59 *p* = 0.000	H = 111.57 *p* = 0.000	H = 111.27 *p* = 0.000	H = 108.88 *p* = 0.000	H = 107.51 *p* = 0.000

H—Kruskal–Wallis test; *p*—probability value.

**Table 2:** Effects of age: 1, 14, 21, 28, and 35 days old for the particular tissues/fat body locations on the activities of CAT, GPx, GST, SOD, and TAC levels.

	CAT	GPx	GST	SOD	TAC
Hemolymph	H = 199.87 *p* = 0.000	H = 195.18 *p* = 0.000	H = 200.60 *p* = 0.000	H = 195.63 *p* = 0.000	H = 195.18 *p* = 0.000
Tergite 3	H = 198.25 *p* = 0.000	H = 190.90 *p* = 0.000	H = 198.75 *p* = 0.000	H = 195.06 *p* = 0.000	H = 204.74 *p* = 0.000
Tergite 5	H = 200.75 *p* = 0.000	H = 192.13 *p* = 0.000	H = 193.69 *p* = 0.000	H = 195.12 *p* = 0.000	H = 204.32 *p* = 0.000
Sternite	H = 200.31 *p* = 0.000	H = 190.40 *p* = 0.000	H = 202.57 *p* = 0.000	H = 200.26 *p* = 0.000	H = 202.79 *p* = 0.000

H—Kruskal–Wallis test; *p*—probability value.

## Data Availability

Data are contained within the article. Upon a justified request of an interested party, they may be made available by the corresponding author.
